# Identification of New Cultivar and Different Provenances of *Dendrocalamus brandisii* (Poaceae: Bambusoideae) Using Simple Sequence Repeats Developed from the Whole Genome

**DOI:** 10.3390/plants13202910

**Published:** 2024-10-17

**Authors:** Ruiman Geng, Junlei Xu, Jutang Jiang, Zhanchao Cheng, Maosheng Sun, Nianhe Xia, Jian Gao

**Affiliations:** 1 Key Laboratory of National Forestry and Grassland Administration/Beijing for Bamboo & Rattan Science and Technology, International Center for Bamboo and Rattan, Beijing 100102, China; gengruiman@icbr.ac.cn (R.G.); xjl@icbr.ac.cn (J.X.); jiangjutangtea@163.com (J.J.); czc@icbr.ac.cn (Z.C.); 2 Institute of Bamboo and Rattan, Southwest Forestry University, Kunming 650224, China; maoshs@imbcams.com.cn; 3 South China Botanical Garden, Chinese Academy of Sciences, Guangzhou 510650, China; nhxia@scbg.ac.cn

**Keywords:** *D. brandisii*, ‘Manxie No.1’, microsatellite development, provenances and cultivar identification, DNA fingerprinting, plant genetic markers, bamboo species identification, genetic diversity analysis, molecular characterization of plants

## Abstract

*Dendrocalamus brandisii* is a high-quality bamboo species that can be used for both bamboo shoots and wood. The nutritional components and flavors of *D. brandisii* from different geographical provenances vary. However, the unique biological characteristics of bamboo render morphological classification methods unsuitable for distinguishing them. Although the new cultivar ‘Manxie No.1’ has significant differences in the branch characteristics and the color of shoot sheaths compared with *D. brandisii*, precise genetic information at the molecular level is still lacking. This study identified 231,789 microsatellite markers based on the whole genome of *D. brandisii* and analyzed their type composition and distribution on chromosomes in detail. Then, using TP-M13-SSR fluorescence-labeling technology, 28 pairs of polymorphic primers were screened to identify the new cultivar ‘Manxie No.1’ and 11 different geographical provenances of *D. brandisii*. We also constructed DNA fingerprinting profiles for them. At the same time, we mapped two polymorphic SSRs to the CDS sequences of two genes of *D. brandisii*, among which SSR497 was mapped to *DhA15G005980.1*, which is related to plant growth and development processes, as well as hormone signal transduction pathways. The specific markers selected in this study can rapidly identify the provenances and the new cultivar of *D. brandisii*, and help to explore candidate genes related to some important traits.

## 1. Introduction

Bamboo is a monocotyledonous plant in the Bambusoideae of the Poaceae [1]. Its enormous regeneration ability and carbon sequestration efficiency make it an astonishingly effective carbon sink and an indispensable plant for mitigating global warming [2]. The three famous species of sweet bamboo are *Dendrocalamus brandisii*, *Dendrocalamus hamiltonii*, and *Dendrocalamus asper*. The main origins of *D. brandisii* are in Southern India, Northeastern India, and Myanmar, and it has been introduced to Southeast Asia. It is mainly distributed in Yunnan Province, China, as well as some South and Southeast Asian countries [3,4]. It has excellent characteristics, such as a high survival rate, fast growth, fast emergence, high yield, and good-tasting bamboo shoots. Therefore, it has become a key bamboo species for shoot development in Yunnan Province, China [5]. Previous studies have shown that there are differences in the nutritional composition and flavor of bamboo shoots from different provenances of *D. brandisii* [6]. Still, they cannot be distinguished by their appearance. The new cultivar ‘Manxie No.1’ is rich in more flavonoids and fewer bitter amino acids, thus having higher nutritional value and better flavor [7]. Although ‘Manxie No.1’ has underdeveloped main branches and significant differences in the color of the shoot sheaths compared to *D. brandisii* (Figure S1), the identification of new cultivars is still difficult to carry out as the shoot sheaths gradually fall off during the growth process, and the property protection of new cultivars is also threatened to some extent. Therefore, identifying the provenances and new cultivars of *D. brandisii* has important practical significance and market application value.

Different from crops, the genetic composition of bamboo is basically wild [8]. Most bamboo has a long flowering cycle and dies after flowering, a special biological characteristic that makes traditional morphological classification based on reproductive characteristics unsuitable for bamboo. This has prompted bamboo taxonomists to pay more attention to the classification of vegetative characters [9,10]. However, there are significant differences in the morphology of bamboo’s vegetative organs, making confusion a prominent issue in bamboo classification, especially in species identification. Overall, bamboo classification mainly relies on vegetative organs that are easily affected by the environment, and the process is cumbersome and complex with low accuracy. The unclear species pedigree and obstacles in cultivar identification are important reasons that restrict the development of the bamboo industry [11]. Currently, there is no unified and authoritative classification system, and bamboo classification remains an important issue in bamboo resource research.

With the development of modern molecular biology technology, molecular marker techniques such as RFLP, RAPD, AFLP, SCAR, SSR, ISSR, etc., have been used for germplasm identification and the phylogenetic determination of bamboo plants [12,13,14,15], which greatly improves the accuracy of classification. SSR, also known as a microsatellite, refers to short DNA sequences with variable tandem repeats of 1–6 bp. SSR is one of the most informative and powerful molecular markers in biology. Due to its high inter-species transferability, co-dominant inheritance, multiple alleles, wide genome coverage, high relative abundance, and simple detection, it has been prioritized for many genetic studies [16,17,18,19,20]. The innovation of capillary electrophoresis technology has enhanced the accuracy of SSR [21]; the combination of these two technologies is popular in cultivar identification and provenance identification.

At present, whole-genome SSR markers have been developed for only a few bamboo species in the genera *Phyllostachys* and *Guadua* and few related genetic analyses in bamboo, such as *Phyllostachys edulis* and *Guadua chacoensis*, have been conducted [15,22]. For the genus *Dendrocalamus*, there are only reports on the development of whole-genome-based SSR markers for *Dendrocalamus strictus* [23], genome survey sequencing-based SSR markers for *Dendrocalamus longispathus* [24], and RNA-seq-based SSR markers for *Dendrocalamus latiflorus* [25], whereas there have been no reports on the development of whole-genome SSR markers for other bamboo species in this genus. According to the existing literature, all studies mainly focus on different bamboo species. However, reports on the same bamboo species from different geographical provenances are scarce. This study was based on the genome of *D. brandisii*. SSR markers covering the whole genome were developed and located in different regions of chromosomes and genes. After the specific primers were obtained via agarose gel electrophoresis, 28 polymorphic primers were further screened using capillary electrophoresis to evaluate the polymorphism potential of the new cultivar ‘Manxie 1’ and 11 different geographical provenances, and their phylogenetic relationships were explained using cluster analysis. A fluorescent labeled TP-M13-SSR system was established to distinguish the new cultivar ‘Manxie No.1’ and different sources of *D. brandisii*, and their DNA fingerprintings were constructed, providing a simple method for their identification and protection.

## 2. Results

### 2.1. Abundance, Frequency, and Characteristics of Microsatellites

**Figure 1:**
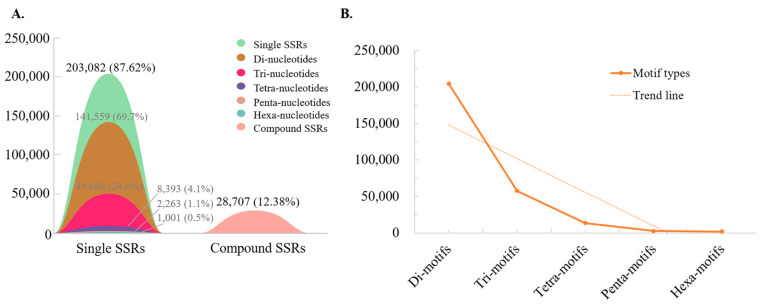
The proportion of different types of microsatellites. (**A**) The number and proportion of different types of microsatellites. The black numbers and corresponding shapes represent the number and proportion of single and composite SSRs in all SSRs. The gray numbers and corresponding shapes represent the number and proportion of different types of perfect SSRs in all perfect SSRs. (**B**) Trends in the number of perfect SSRs with different repetitive motifs.

Based on bioinformatics analysis, we detected 231,789 microsatellites in the 2813.5 Mb genome of *D. brandisii*, of which 231,110 (99.71%) microsatellites were located on chromosomes. The number of perfect SSRs and complex SSRs was 203,082 and 28,707, respectively (Figure 1A), with a microsatellite frequency of 82.4/Mb. In 87.62% of perfect SSRs, the proportion of motifs with different numbers of bases varies. The proportion of microsatellites with dinucleotide repeat motifs is the highest at 69.7%; microsatellites with trinucleotide repeat motifs account for 24.56%, followed by which microsatellites with 4–6 nucleotide repeat motifs account for a smaller proportion, being 4.1%, 1.1%, and 0.5%, respectively (Figure 1A).

The 28,707 compound SSRs identified in this study were composed of 2–78 perfect SSRs and intermediate sequence connections of less than 100 bp. Among them, the number of composite SSRs containing two perfect SSRs was the highest (21,789), accounting for 75.9%, followed by the number of composite SSRs containing three perfect SSRs (4069), accounting for 14.2%. The number of composite SSRs containing 4–78 perfect SSRs was relatively low (1–1119), accounting for 0.003% to 3.899% (Table S1). For compound SSRs containing two perfect SSRs, the length of the connecting sequence is mostly 0 nt (9124), accounting for 41.87%, followed by 1 nt (1305) and 2 nt (1258), accounting for 5.99% and 5.77%, respectively. There are fewer connecting sequences with a length of 3 nt to 100 nt (41–357), accounting for 0.19% to 1.64% (Table S2).

### 2.2. Frequency and Characteristics of Motif Repeat

We identified 280,317 motifs, and the number of motifs with different base numbers decreased with increasing motif length (Figure 1B). The main types of motifs with different base numbers are as follows: the di-nucleotide motifs are mainly AT/AT, AG/CT, and GA/TC; the tri-nucleotide motifs are mainly GCC/GGC, CGC/GCG, and CCG/CGG; the tetra-nucleotide motifs are mainly CATA/TATG, ATAC/GTAT, and TACA/TGTA; the penta-nucleotide motifs are mainly GAGCC/GGCTC, CGAGC/GCTCG, and AAAAG/CTTTT; and the hexa-nucleotide motifs are mainly CATATA/TATATG, ATATAC/GTATAT, and TATACA/TGTATA (Table 1).

**Figure 2:**
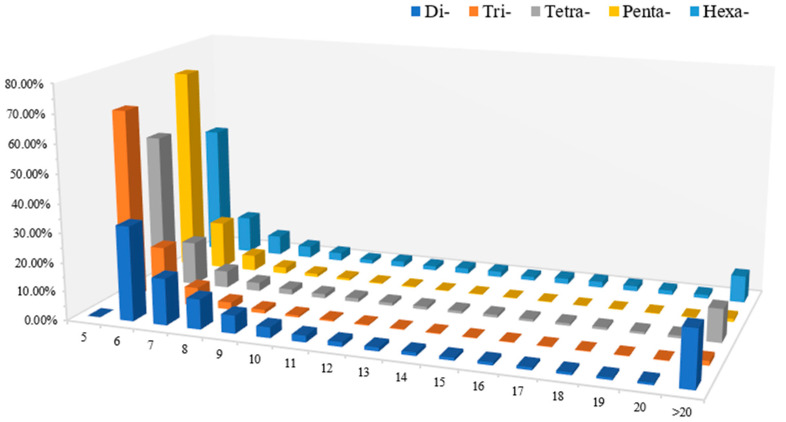
Changes in the content of 2–6 nt motifs with different repetitions. The horizontal axis represents the number of repetitions of 2–6 nt motifs; the vertical axis represents the proportion of a certain type of motif with a certain number of repetitions.

Among the 203,082 perfect SSRs identified in this study, the number of repeats of di-nucleotide motifs ranged from 6 to 187, with those with a repeat frequency of 6 to 8 dominant, accounting for a total of 59.00%. The number of repeats of tri-nucleotide motifs ranged from 5 to 127, with those with a repeat frequency of 5 to 6 dominant, accounting for a total of 85.41%. The number of repeats of tetra-nucleotide motifs ranged from 5 to 571, with those with a repeat frequency of 5 to 6 dominant, accounting for a total of 67.15%, The number of repetitions of the penta-nucleotide motif is 5–72, with those with 5–6 repetitions dominating, accounting for a total of 87.80%. The number of repetitions of the hexa-nucleotide motif is 5–42, with those with 5–6 repetitions dominating, accounting for a total of 58.24% (Figure 2; Table S3).

### 2.3. The Distribution of SSRs in the Genome

**Figure 3:**
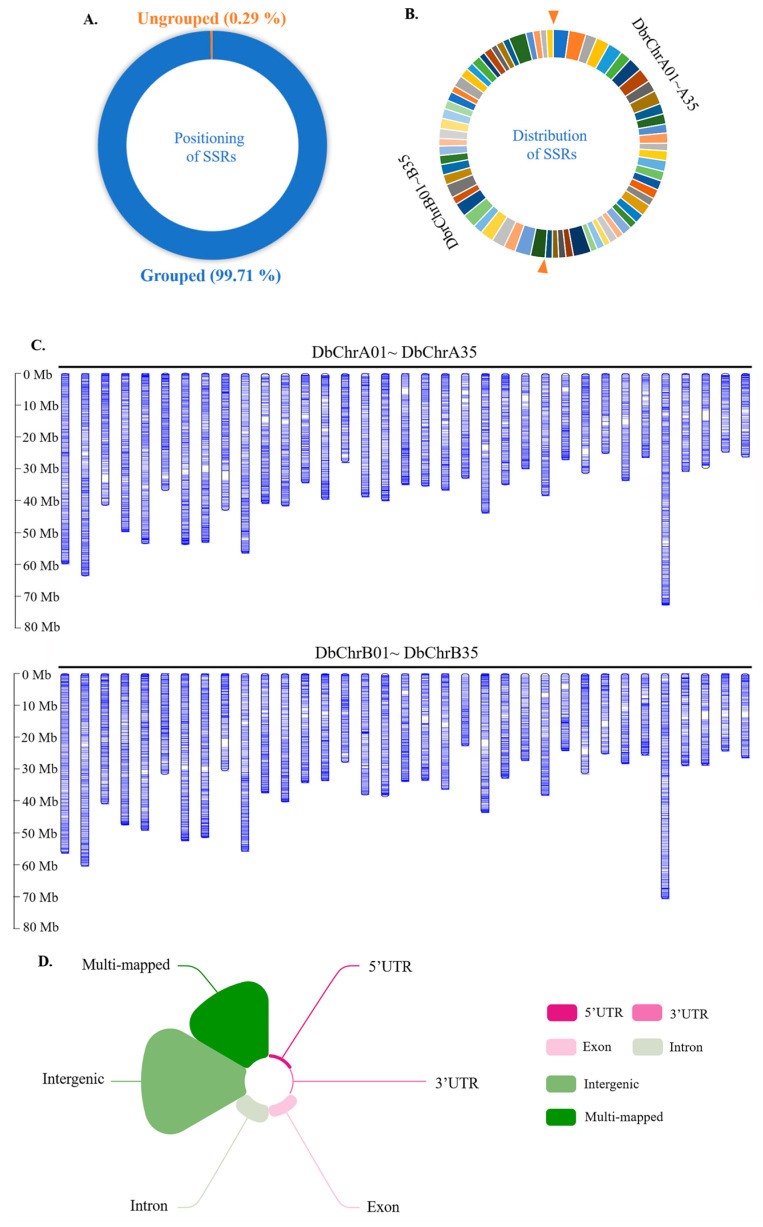
Localization of SSR on the *Dendrocalamus brandisii* chromosome. (**A**) The proportion of SSRs successfully located on chromosomes. (**B**) The proportion of SSR on 70 chromosomes. The right side of the two orange arrows represents the number of SSRs on DbrChrA01–A35 in a clockwise direction, while the left side of the two orange arrows represents the number of SSRs on DbrChrB01–B35 in a clockwise direction. (**C**) Localization of SSRs on the 5’UTR, 3’UTR, exon, intron, intergenic, and multi-mapped *D. brandisii*. (**D**) The distribution of SSR on 70 chromosomes of *D. brandisii*.

Among the 231,789 identified SSRs, 99.71% were evenly distributed on 70 chromosomes of *D. brandisii*, and 0.29% were not successfully localized on chromosomes (Figure 3A–C). Based on the location of SSRs on the genome and genome annotation files, we divided these SSRs into six regions, including 5’UTR, 3’UTR, exons, introns, intergene, and multiple mapping regions. SSRs located in multiple mapping regions are defined as SSRs that can be mapped to two or more regions simultaneously. As shown in Figure 3D, through the analysis of SSRs, the results show that SSRs are generally mapped to the intergenic region, and multiple mapping SSRs also account for a large proportion, followed by SSRs mapped to introns and exons, while SSRs mapped to 5’UTR and 3’UTR are relatively few in number.

### 2.4. Obtain 28 Pairs of Polymorphic Primers and Perform Gene Mapping on 2 of Them

We utilized the Primer3_core program for Perl language-designed batch primers for all microsatellite flank sequences, resulting in 20,228 pairs of primers. Then, we randomly selected 800 pairs of primers for synthesis, and combined PCR and agarose gel electrophoresis to preliminarily screen them; the results showed that 433 pairs of primers could amplify at least one band (Figure S2). We selected 72 pairs of specific primers that could only amplify one band to conduct TP-M13-SSR PCR amplification on ‘Manxie No.1’ and 11 different geographical provenances of *D. brandisii* materials. The capillary electrophoresis results showed that the results for the three individuals within each provenance population were consistent, so any one of the samples could be selected as a representative sample for that provenance population. And 28 pairs of primers (Table S4) exhibited polymorphism, with adjacent alleles of each marker differing by multiples of the repeat unit length, indicating that these 28 polymorphic SSR markers are effective and have conservation and transferability across cultivars and provenances.

**Figure 4:**
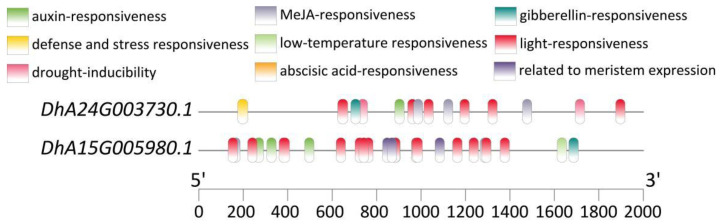
Diagram of cis-acting elements in the promoters of *DhA24G003730.1* and *DhA15G005980.1*.

In addition, we performed gene mapping on two SSRs (SSR236, SSR497) located on exons, which corresponded to *DhA24G003730.1* and *DhA15G005980.1*, respectively. Through homology alignment in the NCBI database, it was revealed that *DhA24G003730.1* encodes the dihydrolipoyllysine-residue acetyltransferase component 4 of the pyruvate dehydrogenase complex in chloroplasts. It is postulated to participate in the maintenance and renewal of the membrane structure within the chloroplast. *DhA15G005980.1* is a *DIVARICATA* transcription factor, which belongs to the *MYB* family. This gene is associated with various aspects of plant growth and development, and participates in regulating hormone signal transduction, non-biological stress resistance, and other processes. Notably, the promoters of both genes harbor cis-acting elements responsive to light, auxin, gibberellin, and methyl jasmonate (Figure 4). These elements likely play crucial roles in the transcriptional regulation of these genes in response to diverse environmental and hormonal cues, thereby modulating their physiological functions within the plant.

### 2.5. Genetic Diversity Analysis of ‘Manxie No.1’ and 11 Different Geographical Provenances of D. brandisii Materials Based on 28 Polymorphic SSRs

Genetic diversity analysis was conducted on ‘Manxie No.1’ and 11 different geographical provenances of *D. brandisii* representative materials using 28 polymorphic SSR markers. A total of 28 polymorphic SSR markers amplified 81 alleles, with an average of 2.8929 alleles per marker. Among them, SSR394 amplified the largest number of alleles (Na = 6). In contrast, 14 markers, such as SSR52 and SSR18, amplified the fewest alleles (Na = 2). The range of Ne was 1.1803 to 4.0000, with an average of 2.0281 and an average Ho value of 0.5268, which was higher than the average He value of 0.4732. The average value of I was 0.7600 (range of 0.2868–1.4735). The PIC range of polymorphic information was 0.1411–0.7078, with an average value of 0.3863. There were seven pairs of primers with PIC < 0.25, indicating low polymorphism. The PIC of six primer pairs was greater than 0.5, which indicates that they possess high polymorphism (Table 2). The correlation graph between Na and PIC values shows that. as the number of alleles increased, the polymorphism of specific loci increased (Figure S3). The genetic parameters calculated based on 28 SSR markers indicate that there was a moderate degree of genetic diversity and variation rate between 11 different geographical provenances of *D. brandisii* and ‘Manxie No.1’.

### 2.6. Cluster Analysis of 1 ‘Manxie No.1’ and 11 Different Geographical Provenances of D. brandisii Materials Based on 28 SSR Markers

**Figure 5:**
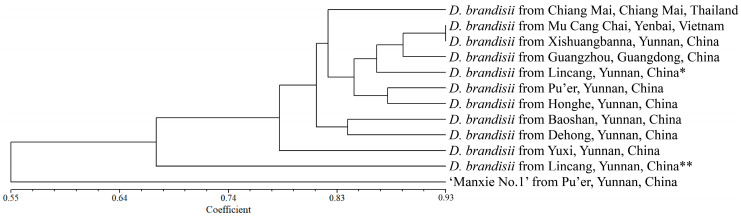
Cluster analysis of 12 materials based on 28 SSR markers. * represents Cangyuan County, Lincang City, Yunnan Province, China, and ** represents Linxiang District, Lincang City, Yunnan Province, China.

Based on NTSYS v.2.10e software, the genetic similarity coefficient was calculated using the SM coefficient, and a UPGMA tree was constructed based on the genetic similarity coefficient. Similarly, as reported in Result 2.5, we conducted cluster analysis using a representative sample from each population. The analysis revealed that the new variety ‘Manxie No. 1’ exhibited a certain degree of genetic divergence compared with *D. brandisii*, with a mean genetic similarity coefficient of 0.3823, indicating a relatively distant genetic relationship. This finding is congruent with the fact that ‘Manxie No. 1’ is a superior variety obtained through artificial selection. In the case of *D. brandisii*, the samples from Lincang** (Linxiang District) and Yuxi City in Yunnan Province, China, demonstrated a more distant genetic relationship with the other nine provenances. The remaining nine provenances of *D. brandisii* could be approximately divided into two groups based on the clustering tree. The samples from Baoshan City and Dehong Dai and Jingpo Autonomous Prefecture (abbreviated as ‘Dehong’ in the following text), which are geographically adjacent to each other in Yunnan Province, China, constituted one group, with a genetic similarity coefficient of 0.7292 between them. In the other group, the samples from Chiang Mai, Thailand, formed a separate subgroup, while those from Mu Cang Chai County, Vietnam, Xishuangbanna Dai Autonomous Prefecture (the following text is abbreviated as ‘Xishuangbanna’) and Lincang* (Cangyuan County) in Yunnan Province, China, and Guangzhou in Guangdong Province, China, were grouped together. Among them, the samples from Mu Cang Chai County, Vietnam, and Xishuangbanna, China, exhibited the closest genetic relationship, with a genetic similarity coefficient of 0.8667. The samples from Pu’er City and Honghe Hani, and Yi Autonomous Prefecture (abbreviated as ‘Honghe’ in the following text), which are geographically adjacent to each other in Yunnan Province, China, constituted the third subgroup, with a genetic similarity coefficient of 0.7826 (Figure 5; Table S5). The clustering results indicate that there was a certain correlation between the genetic relationships of different geographical provenances of *D. brandisii* and their geographical distribution. However, it was not completely related to their geographical distribution. Further verification of the accuracy of the UPGMA tree was carried out through the Cophenetic values subroutine in the clustering program and the matrix comparison plot subroutine in the graphics program. The matrix correlation r value of the correlation test was 0.9424, proving that the reliability of the UPGMA tree was very high. 

*D. brandisii*, *Dendrocalamus hamiltonii,* and *Dendrocalamus asper* are all part of the *Dendrocalamus* genus and are known as the world’s three largest bamboo plants with sweet bamboo shoots. We conducted cluster analysis on 2 samples of *D. asper*, 1 sample of *D. hamiltonii*, and the aforementioned 12 materials. The results showed that the genetic relationship between *D. hamiltonii* and *D. brandisii* was closer than that between *D. asper* and *D. brandisii* (Figure S4), and these three types of sweet dragon bamboo could each cluster into one branch. The clustering results were consistent with existing classifications, confirming the applicability and effectiveness of the microsatellite markers identified in this study.

### 2.7. SSR Core Primers and DNA Fingerprinting of 1 ‘Manxie No.1’ and 11 Different Geographical Provenances of D. brandisii Materials

**Figure 6:**
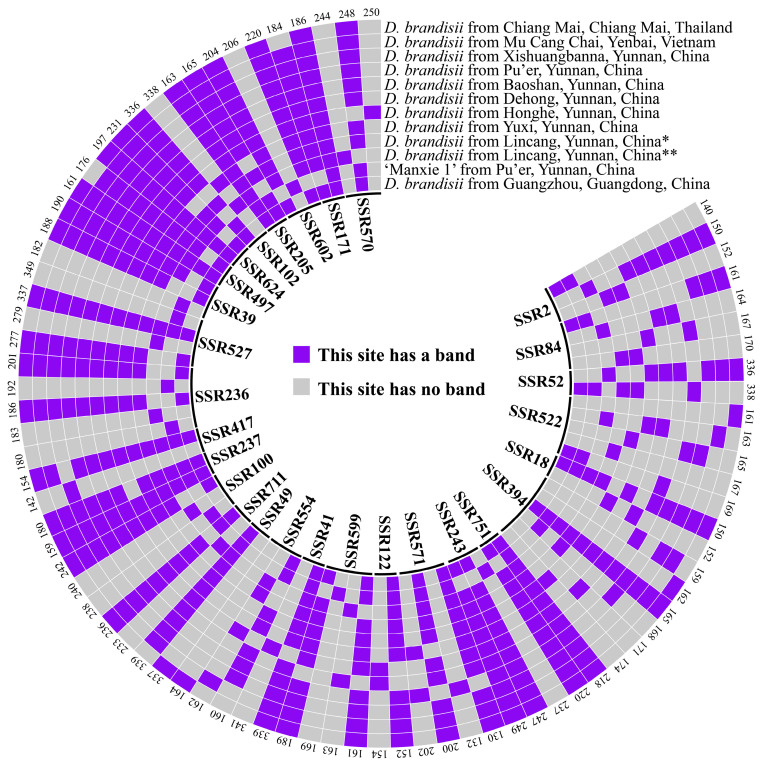
DNA fingerprinting of 12 materials constructed based on 28 pairs of SSR primers. On the inner circle of the image is the name of the 28 SSRs. The number on the outer circle of the image represents the size of all fragments that the corresponding SSR can amplify. Blue and gray, respectively, represent the presence or absence of fragments. * represents Cangyuan County, Lincang City, Yunnan Province, China, and ** represents Linxiang District, Lincang City, Yunnan Province, China.

In this study, primer SSR394 exhibited the highest number of producible bands (six bands), followed by SSR522 (five bands). These bands could be respectively assembled into five distinct banding patterns, which were utilized to discriminate five different materials. When the primer combinations of SSR522 and SSR394 were employed in conjunction with any one of SSR599, SSR100, SSR236, SSR527, or SSR39, a complete differentiation of the twelve materials was achieved. Consequently, these five combinations were each designated as a set of core primers. Moreover, four additional sets of primers were also authenticated as core primers. All nine sets of core primers are enumerated in Table S8. The DNA fingerprinting can be found in Figure 6 and Table S6. 

## 3. Discussion

SSRs are considered popular markers for genetic diversity evaluation, germplasm resource identification, purity analysis, and genetic mapping in various organisms due to their high polymorphism, high information content, codominance, and ability to detect alleles at specific loci in a single organism. The rapid development of next-generation sequencing technology has made SSR whole-genome identification easier and more comprehensive [26,27,28,29,30]. There are studies indicating that TP-M13-SSR fluorescence labeling may not be suitable for the cultivar identification of large-genome species [31]. However, the results of this study indicate that TP-M13-SSR fluorescent labeling can be used for DNA fingerprint construction and cultivar and provenances identification of *D. brandisii* with a large genome, and the system has high throughput, high sensitivity, and high accuracy, making it an effective method for fingerprint identification and the identification of asexual reproduction of *D. brandisii*.

### 3.1. SSR Marker Features Developed Based on the Whole Genome of D. brandisii

This study conducted genome-wide SSR identification and analysis on *D. brandisii*. A total of 231,789 microsatellites were detected from the genome of *D. brandisii* at 2813.5 Mb, with a microsatellite frequency of 82.4/Mb. The number of microsatellites was much larger than that of smaller genomes such as *Arabidopsis thaliana* (119.7 Mb) and *Oryza sativa* (374.5 Mb). However, the microsatellite frequency was much lower than both (135.5/Mb in *A. thaliana* and 165.5/Mb in *O. sativa*). Compared with the smaller genomes of *Phyllostachys edulis* (2051.7 Mb, 127,593, 62.2/Mb) and *Zea mays* (2066.4 Mb, 107,742, 52.1/Mb), the number of microsatellites is about twice that of the two, and the frequency of microsatellites is also higher [22]. This is inconsistent with the previous research findings that species with larger gene groups have more SSRs but a lower SSR density [32,33]. The number and density of microsatellites may not be related to the genome size.

Generally speaking, there are a large number of di-nucleotide and tri-nucleotide repeats, while the abundance of tetra-nucleotide to hexa-nucleotide repeat sequences is relatively low in the eukaryotic genome [34,35]. Among them, di-nucleotide repeat sequences are considered an indispensable resource due to their highest mutation rate and abundant quantity [36]. In this study, 94.3% of microsatellite repeat motifs were di-nucleotide and tri-nucleotide repeats, of which nearly 70% were di-nucleotide repeats. However, tetra-nucleotide, penta-nucleotide, and hexa-nucleotide repeats only accounted for a very small portion (5.7%). Similar distributions of repeat motifs were also observed in other Poaceous species, such as *Ph. edulis*, *Z. mays*, *O. sativa*, *B. distachyon,* and *S. bicolor*, and di-nucleotide and tri-nucleotide repeats still dominate [22]. This is consistent with the usual view that the abundance of microsatellites decreases with increasing repetition length [37,38].

Previous studies have shown that the GC content is higher in animal genomes, while the AT content is higher in plant genomes [39,40]. The data in this study show that the AT content in *D. brandisii* is also higher than the GC content, which is consistent with other bamboo species such as *Guadua chacoensis*, *Dendrocalamus latiforus*, etc. [15,41], and is also consistent with other species such as *A. thaliana* and *S. bicolor* [29]. Among many species, AG/CT motifs have the highest proportion, including Poaceae plants such as *Ph. edulis*, *Z. mays*, *O. sativa*, *B. distachyon,* and *S. bicolor* and non-Poaceae plants such as *Epimedium sagittatum* [22,42]. However, in our study, AG/CT motifs (19.44%) were the type with the highest proportion in microsatellites except for AT/AT motifs (35.11%), similar to *S. bicolor* and *A. thaliana* [22]. Previous studies have shown that in plants, TC repeat sequences are typically present in the transcriptional region and frequently appear in 5’UTRs. CT microsatellites in 5’UTRs may be involved in antisense transcription and play a role in gene regulation [43]. This suggests that approximately 1/5 of the microsatellite markers developed in this study may be involved in the antisense transcription of *D. brandisii*, thereby regulating the function of related genes. Monocotyledons have more abundant tri-nucleotide repeat sequences rich in GC than dicotyledonous plants [44]. Monocotyledons such as *O. sativa*, *B. distachyon*, and *S. bicolor* have the highest content of CCG/CGG, while *Ph. edulis* has the second highest content of CCG/CGG [22]. However, dicotyledonous plants such as *A. thaliana* and *Glycine max* rarely exhibit CCG/CGG [44]. In this study, GCC/GGC was the type with the highest proportion of tri-nucleotide repeat motifs in *D. brandisii*, accounting for 11.17%, followed by CGC/GCG (8.93%) and CCG/CGG (8.01%), which is consistent with this conclusion. Tetra-nucleotide, penta-nucleotide, and hexa-nucleotide repeat sequences, as in most plants, have a lower frequency.

### 3.2. Genetic Diversity within Bamboo Populations and Determination of Representative Samples for Various Populations of D. brandisii

This study fully considered the typicality and representativeness when selecting samples. We conducted a detailed investigation and analysis of the sample collection site to ensure that the environmental conditions and bamboo growth status of the site can represent the overall situation of the entire study area. At the same time, as bamboo mainly reproduces asexually through rhizomes, in order to avoid duplicate sampling, different samples selected from the same population come from noncontinuous bamboo forests with differences in latitude, longitude, and altitude, ensuring that duplicate samples have relatively independent genetic sources. This precisely proves that the sample we have chosen is more representative. By maintaining a larger sampling distance, the likelihood of duplicate samples being disturbed by the same environment can be reduced, allowing them to reflect the main characteristics of the entire population [45]. The samples we selected showed consistent results under these different conditions, further indicating that they can represent the overall characteristics of geographical provenances to some extent.

Previous studies have shown that there is indeed variation within populations of different provenances of *D. brandisii*, but this variation is very low [46]. This study showed that the Hs value of allelic marker diversity within populations of different provenance of *D. brandisii* was 0.0382 ± 0.0032; the mean values of the observed number of alleles (Na), the effective number of alleles (Ne), Shannon’s information index (I), and expected heterozygosity (He) among different individuals within various populations are 1.1338 ± 0.2978, 1.0906 ± 0.2168, 0.0742 ± 0.1704, and 0.0507 ± 0.1184. It is generally believed that genetic diversity is low when the Hs value is less than 0.1, the Na value is less than 2, the Ne value is close to or less than 1, the I value is less than 0.5, and the He value is less than 0.3. Given this, it can be seen that these values are relatively low, indicating limited genetic variation and low levels of genetic diversity among different individuals within the same population of *D. brandisii*. In our study, we selected three independent samples for each provenance population. Only when the results are consistent can we select one sample as a representative sample. If the results are inconsistent, additional samples need to be added for supplementary confirmation. The repeated individuals selected in the study are located at a certain distance and altitude from each other, and the possibility that they are all variant individuals and that the results of the variation reflected in the 28 polymorphic SSR markers are completely consistent is minimal. Therefore, we believe that consistent results from three replicates are sufficient to support the idea of selecting one as a representative sample for preliminary cluster analysis.

In future research, we will continue to improve our sampling methods to further enhance the typicality and representativeness of samples. Nonetheless, we are also aware that increasing the number of samples for each population would be preferable. We plan to expand the sample size, explore more diverse *D. brandisii* habitats, and conduct more in-depth genetic analysis to capture a more comprehensive picture of its populations and uncover the factors that contribute to limited genetic diversity. This will help us develop more targeted conservation strategies and management plans for *D. brandisii* resources. Moreover, we will explore innovative research methods and technologies to better understand the genetic characteristics and evolutionary history of *D. brandisii*. This could include the use of advanced genomic sequencing techniques and computational models to analyze large-scale genetic data. By doing so, we hope to make contributions to the field of *D. brandisii* research and conservation and promote the sustainable use and protection of these important resources.

### 3.3. Genetic Diversity and Clustering of 1 ‘Manxie No.1’ and 11 Different Geographical Provenances of D. brandisii

Due to the unique reproductive characteristics of bamboo, the classification problem has been troubling researchers. Molecular markers are genetic markers based on nucleotide sequence differences between individuals or populations, which intuitively and clearly reflect biological diversity at the DNA level. They are suitable and effective for identifying different plant genera, species, and underspecies. Domestic and foreign scholars have used isoenzyme labeling and molecular marker methods such as the RFLP (Restriction Fragment Length Polymer), RAPD (Random Amplified Polymer DNA), AFLP (Amplified Fragment Length Polymer), ITS (Internal Transcribed Spacer), and SSR (Simple Sequence Repeat) to study the genetic diversity and phylogenetic relationships between bamboo species and genera [30,47,48,49,50]. However, due to the characteristics of asexual reproduction of bamboo plants, there is little research on the genetic diversity of bamboo plants at the subspecies level. We used co-dominant SSR molecular marker technology to successfully identify different provenances of *D. brandisii* and the new cultivar ‘Manxie No.1’. Moreover, we constructed a DNA fingerprinting of 12 materials based on 28 SSR molecular markers to reflect their differences at the DNA level. This provides a molecular basis for the effective identification of different provenance of *D. brandisii* and the new cultivar ‘Manxie No.1’.

At present, there is relatively scarce information on the genetic diversity and population structure of bamboo germplasm resources. Due to the asexual reproduction of bamboo through rhizomes, it has low intra-species genetic diversity and genetic differences between populations [51]. Therefore, screening for markers with relatively high abundance and polymorphism for bamboo may be very difficult. Abreu developed and screened 7 polymorphic microsatellite markers for *Aulonemia aristulata* [52], and Vieira developed and screened 16 polymorphic microsatellite markers for the tropical woody bamboo Tribe Bambusae (Poaceae: Bambusoideae) [53]. Weixin developed and screened 20 polymorphic microsatellite markers for *Ph. Edulis* [32]. In this study, based on the ability of agarose gel to separate DNA fragments, 72 pairs of specific primers that can amplify only 1 band were initially screened from 800 pairs of primers, thus reducing the complexity and uncertainty in subsequent analyses. Then, based on capillary electrophoresis with higher resolution and sensitivity, 28 pairs of polymorphic primers were further selected from 72 pairs of specific primers that can amplify DNA fragments of different lengths in different samples. The differences in fragment lengths reflect the different number of motif repeats in SSR loci, thereby reflecting genetic differences between samples. Using these 28 pairs of polymorphic SSR primers, genetic diversity analysis was conducted on 12 samples (‘Manxie No.1’ and 11 different geographical provenances of *D. brandisii*). The PIC value takes into account the frequency and number of alleles and can comprehensively reflect the degree of polymorphism of the locus. For example, at an SSR locus, if there are multiple alleles and their frequency distribution in the sample is relatively uniform, the PIC value of the locus will be higher, indicating that the locus has high genetic diversity. In this study, the average PIC value of these 12 samples was 0.3863, ranging from 0.1411 to 0.7078, indicating that these samples had a moderate level of genetic diversity. This result provides important information for us to understand the genetic background of these samples. The moderate level of genetic diversity means that the samples had both a certain degree of genetic variability and relative stability. This genetic diversity may be due to the samples coming from different geographical provenances, experiencing different environmental choices, and genetic drift. On average, compared with other bamboo species using SSR molecular markers, the genetic diversity level of sweet dragon bamboo is higher than those of *Guadua inermis* and *Ph. edulis* [51,54] but lower than those of *G. angustifolia* in the Colombian coffee eco-region and *Kuruna debilis* [55,56].

Cluster analysis shows that the genetic diversity of *D. brandisii* and ‘Manxie No.1’ exhibits extensive variation. ‘Manxie No.1’ can be distinguished from different geographical provenances of *D. brandisii* and *D. hamiltonii,* and *D. asper* can also be distinguished from *D. brandisii*. Multiple primer combinations can distinguish 12 sweet dragon bamboo materials. However, clustering analysis shows that the genetic relationship between ‘Manxie No.1’ and *D. brandisii* is farther than that between *D. hamiltonii*, *D. asper* and *D. brandisii*. The possible reason for this result is that the number of microsatellite molecular markers is insufficient, leading to clustering bias. This situation can be improved by further screening polymorphic markers.

### 3.4. Identification of Candidate Genes Based on Polymorphic SSRs

This study performed gene localization on SSR236 and SSR497, and found that they were located in the exon regions of DhA24G003730.1 and DhA15G005980.1, respectively. DhA24G003730.1 encodes the dihydrothiolysine residue acetyltransferase component 4 of the pyruvate dehydrogenase complex in chloroplasts. A well-functioning pyruvate dehydrogenase complex is necessary for lipid synthesis, providing the necessary energy and material basis for the biosynthesis, maintenance, and renewal of thylakoid and chloroplast membranes [57,58,59,60], thereby ensuring the normal biological functions of chloroplasts. DhA15G005980.1, being a DIVARICATA transcription factor of the MYB family, has been demonstrated to play a significant and multifaceted role in plant growth and development, signal transduction, as well as in response to abiotic stresses. For instance, this transcription factor has been identified as essential for the determination of ventral petal identity in Antirrhinum majus [61], and it plays a pivotal role in establishing the floral dorsoventral asymmetry [62]. Moreover, it can negatively regulate salt stress in A. thaliana by integrating abscisic acid (ABA) signaling pathways [63]. The presence of cis-acting elements responsive to light, auxin, gibberellin, and methyl jasmonate within the promoters of both genes represents an intriguing discovery. These cis-acting elements are likely to act as regulatory switches, enabling the genes to respond to changes in the external and internal environment. In conclusion, the successful localization of SSR236 and SSR497 within DhA24G003730.1 and DhA15G005980.1, respectively, has opened up new avenues for exploring the chloroplast function, growth and development, plant hormone signal transduction, and resistance to abiotic stress in *D. brandisii*. 

It should be noted that our discussion here is purely speculative. We do not claim any functional correlation between the SSRs and traits at present. Further research is needed to explore any potential functional correlations between these SSRs and specific traits in D. brandisii. For example, future studies could investigate how the presence of these SSRs might affect the phenotypic characteristics of the plant under different environmental conditions.

## 4. Materials and Methods

### 4.1. Plant Materials, Genomic DNA Isolation, and Detection

**Figure 7:**
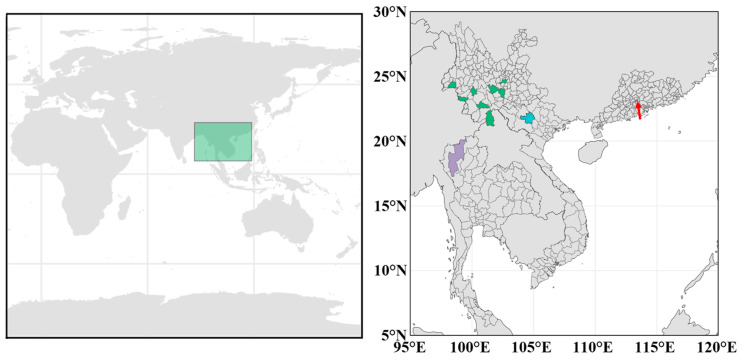
Sampling site labeling diagram for 11 samples of *D. brandisii* and 1 sample of ‘Manxie No.1’. The figure above shows the location of the sampling sites on a world map. In the figure below, green, red, blue, and purple represent the sampling sites in Yunnan Province, China; Guangdong Province, China; Yenbai Province, Vietnam; and Chiang Mai Province, Thailand.

A new cultivar, ‘Manxie No.1’, and 11 different geographical provenances of *D. brandisii* materials were obtained from the main distribution areas of *D. brandisii* in China, Thailand, and Vietnam (Figure 7). There is a certain distance between the duplicate individuals of each provenance population, and they are distributed in discontinuous bamboo forests with different latitudes, longitudes, or altitudes to ensure that the duplicate samples have relatively independent genetic sources and can reflect the main characteristics of the entire germplasm population [45]. Finally, these fresh leaves were collected in a sampling bag containing a discolored silicone desiccant and were stored in a −80 °C refrigerator for future use.

Genomic DNA was extracted from leaf samples of 3 individuals in each population using a slightly improved cetyltrimethylammonium bromide (CTAB) method [64]. NanoDrop one (Thermo Fisher Scientific, Waltham, MA, USA) was used to determine the DNA concentration, and 1.0% agarose gel electrophoresis was used to detect the DNA quality.

### 4.2. Microsatellite Identification and Primer Design

All microsatellites in the entire genome sequence of *D. brandisii* (the genome assembled in reference [7] (https://doi.org/10.1111/jipb.13592 (accessed on 10 December 2023))) were identified using the Perl language platform-based microsatellite identification tool (MISA) (http://pgrc.ipk-gatersleben.de/misa/ (accessed on 6 January 2024)) [65]. The parameter settings are as follows: the di-nucleotide motif should be repeated at least 6 times, and the tri-nucleotide, tetra-nucleotide, penta-nucleotide, and hexa-nucleotide motifs should be repeated at least 5 times. When the sequence length between two simple sequence repeats (SSRs) is less than 100 bp, it is considered a composite SSR [66]. MISA is still used to analyze the number of motifs, repetitions, the number, length, and starting and ending positions of microsatellites with different repeat types on the genome.

The Primer3_core program (https://github.com/primer3-org/primer3 (accessed on 20 January 2024)) suitable for Perl language is used for batch primer design of microsatellite flanking sequences. The main parameter settings are as follows: (i) the primer length is 18–25 bp, (ii) Tm value is 57 °C–62 °C, (iii) the size of the target PCR product is 120–400 bp, and (iv) the GC-content is 40–60%.

### 4.3. Screening for Specificity and Polymorphism of Primers

We randomly selected 800 pairs of SSR primers covering 70 chromosomes for synthesis and used ‘Manxie No.1’ genomic DNA as a template for PCR amplification. When the size of the agarose gel electrophoresis band of the PCR amplification product met the expectation, it could be identified as a specific primer.

Subsequently, TP-M13-SSR PCR was performed using genomic DNA from different geographical provenances of *D. brandisii* and the new cultivar ‘Manxie No.1’ as templates, and the obtained specific primers were further screened for polymorphism. All PCR and electrophoresis were evaluated for consistency and reproducibility of results through multiple repeated experiments. This method consists of three primers: a forward primer with an 18 bp M13 tail sequence (5’–TGTAAAAACGACGGCCAGT–3’) added at the 5’ end, an M13 universal forward primer with a fluorescent group (6-carboxy-X-rhodamine (ROX, red), hexachloro-6-carboxy-flurrescine (HEX, yellow), tetrachloro-6-carboxy-flurrescine (TET, green) or 6-carboxy-flurrescine (FAM, blue)), and a normal specific reverse primer. The PCR system and reaction program adopt a two-step method, as detailed in Table S7. Fluorescence capillary electrophoresis SSR analyzed PCR end-products on the ABI 3730xl DNA analyzer (Thermo Fisher Scientific, USA), and the results were analyzed using Genemarker v2.2.0 software (https://softgenetics.com/products/genemarker/ (accessed on 22 May 2024)) [67]. Here, due to the relatively limited genetic variation and low level of genetic diversity among different individuals within the same provenance population of *D. brandisii* [46], we analyzed and read the results of three individuals in each provenance population. If the three results are completely consistent, we selected any one of them as the representative sample of the provenance population. Otherwise, repeat individuals will be added for further validation to determine representative samples for genetic diversity analysis and cluster analysis.

### 4.4. Data Analyses and Fingerprint Construction

POPGEN32 v1.32 software [68] was used to calculate Nei’s diversity index (Nei’s), Shannon’s information index (I), average observed heterozygosity (HO), and expected heterozygosity (He). PowerMarker v. 3.25 software [69] was used to calculate polymorphism information content (PIC), and NTSYS v. 2.10e software [70] was used to calculate genetic distance and plot the UPGMA cluster tree. Other data analyses and fingerprint construction were completed in Excel 2019.

## 5. Conclusions

Based on the identification of a large number of SSRs from the genome of *D. brandisii*, 28 polymorphic microsatellite markers were screened and validated to quickly and accurately distinguish the new variety ‘Manxie No.1’ and *D. brandisii* from different geographical provenances. These markers help in understanding the distribution of genetic diversity and achieving the sustainable use of resources. At the same time, it provides reliable technical means for selecting varieties with excellent traits in horticultural production. Two polymorphic SSRs were mapped to the genes of *D. brandisii*. This provides candidate genes for studying its chloroplast function, growth and development, plant hormone signal transduction, resistance to abiotic stress, and other aspects. The identification technology based on microsatellite markers can be used as a part of product quality standards to standardize the production and circulation of horticultural products and improve the standardization and specialization level of the entire industry.

## Figures and Tables

**Table 1:** The number of different types of motifs.

Types	Motif Types	Number	%
P2	GA/TC	27,515	19.44%
	AG/CT	34,419	24.31%
	AT/AT	49,705	35.11%
P3	CCG/CGG	3992	8.01%
	CGC/GCG	4451	8.93%
	GCC/GGC	5568	11.17%
P4	TACA/TGTA	506	6.03%
	ATAC/GTAT	618	7.36%
	CATA/TATG	860	10.25%
P5	AAAAG/CTTTT	94	4.15%
	CGAGC/GCTCG	94	4.15%
	GAGCC/GGCTC	105	4.64
P6	TATACA/TGTATA	57	5.69%
	ATATAC/GTATAT	65	6.49%
	CATATA/TATATG	67	6.69%

**Table 2:** Polymorphism indicators of 12 important materials were obtained using 28 SSR markers.

Primer ID	Na ^1^	Ne ^2^	I ^3^	Ho ^4^	He ^5^	PIC ^6^
SSR2	3.0000	2.0000	0.8676	0.4783	0.5217	0.4491
SSR84	4.0000	3.4286	1.3086	0.2609	0.7391	0.6589
SSR52	2.0000	1.9459	0.6792	0.4928	0.5072	0.3680
SSR522	5.0000	4.0000	1.4735	0.2174	0.7826	0.7078
SSR18	2.0000	1.3846	0.4506	0.7101	0.2899	0.2392
SSR394	6.0000	2.3415	1.2109	0.4022	0.5978	0.5436
SSR751	2.0000	1.9862	0.6897	0.4819	0.5181	0.3733
SSR243	3.0000	2.3415	0.9222	0.4022	0.5978	0.4788
SSR571	4.0000	2.5487	1.0618	0.3659	0.6341	0.5280
SSR122	2.0000	1.3846	0.4506	0.7101	0.2899	0.2392
SSR599	4.0000	2.7429	1.1219	0.3370	0.6630	0.5630
SSR41	2.0000	1.1803	0.2868	0.8406	0.1594	0.1411
SSR554	3.0000	2.1818	0.8877	0.4348	0.5652	0.4598
SSR49	2.0000	1.1803	0.2868	0.8406	0.1594	0.1411
SSR711	2.0000	1.1803	0.2868	0.8406	0.1594	0.1411
SSR100	3.0000	1.4118	0.5661	0.6957	0.3043	0.2723
SSR237	2.0000	1.9862	0.6897	0.4819	0.5181	0.3733
SSR417	2.0000	1.1803	0.2868	0.8406	0.1594	0.1411
SSR236	5.0000	2.7961	1.2015	0.3297	0.6703	0.5748
SSR527	4.0000	2.3415	0.9762	0.4022	0.5978	0.4832
SSR39	3.0000	2.3226	0.9184	0.4058	0.5942	0.4768
SSR497	2.0000	1.9459	0.6792	0.4928	0.5072	0.3680
SSR624	2.0000	1.9459	0.6792	0.4928	0.5072	0.3680
SSR102	2.0000	1.3846	0.4506	0.7101	0.2899	0.2392
SSR205	2.0000	1.9459	0.6792	0.4928	0.5072	0.3680
SSR602	3.0000	2.3415	0.9222	0.4022	0.5978	0.4788
SSR171	2.0000	1.9459	0.6792	0.4928	0.5072	0.3680
SSR570	3.0000	1.4118	0.5661	0.6957	0.3043	0.2723
Mean	2.8929	2.0281	0.7600	0.5268	0.4732	0.3863
St.	1.1333	0.6842	0.3268	0.1842	0.1842	0.1571

^1^ Observed number of alleles. ^2^ Effective number of alleles. ^3^ Shannon’s information index. ^4^ Observed heterozygosity. ^5^ Expected heterozygosity. ^6^ Polymorphic information content.

## Data Availability

All data are included in the manuscript.

## Supplementary Materials

The following supporting information can be downloaded at: https://www.mdpi.com/article/10.3390/plants13202910/s1, Figure S1: The difference in shoot sheath color and the number of main branches between ‘Manxie No.1’ and *D. brandisii*. On the left is *D. brandisii*, and on the right is ‘Manchier No.1’. (A) The color difference in shoot sheaths between the two. (B) The color difference in the number of branches between the two. Figure S2: Preliminary screening results of agarose gel electrophoresis with some primers. Figure S3: The correlation between alleles and PIC values. Figure S4: Cluster analysis of 15 sweet dragon bamboo germplasms based on SSR markers. Table S1: The number of perfect SSRs contained in compound SSRs and their respective proportions. Table S2: Connection sequence length of compound SSRs containing two perfect SSRs. Table S3: The number of motif repetitions of various types of perfect SSRs. Table S4: Information on 28 pairs of polymorphic primers. Table S5: Genetic similarity coefficients between every two of the 12 materials. Table S6: DNA fingerprint information of the 12 materials. Table S7: The PCR system and PCR program used in the TP-M13-SSR PCR method. Table S8: Nine sets of core primers for 12 materials.
