# Correction: Geng et al. Identification of New Cultivar and Different Provenances of *Dendrocalamus brandisii* (Poaceae: Bambusoideae) Using Simple Sequence Repeats Developed from the Whole Genome. *Plants* 2024, *13*, 2910

**DOI:** 10.3390/plants14101416

**Published:** 2025-05-09

**Authors:** Ruiman Geng, Junlei Xu, Jutang Jiang, Zhanchao Cheng, Maosheng Sun, Nianhe Xia, Jian Gao

**Affiliations:** 1Key Laboratory of National Forestry and Grassland Administration/Beijing for Bamboo & Rattan Science and Technology, International Center for Bamboo and Rattan, Beijing 100102, China; gengruiman@icbr.ac.cn (R.G.); xjl@icbr.ac.cn (J.X.); jiangjutangtea@163.com (J.J.); czc@icbr.ac.cn (Z.C.); 2Institute of Bamboo and Rattan, Southwest Forestry University, Kunming 650224, China; maoshs@imbcams.com.cn; 3South China Botanical Garden, Chinese Academy of Sciences, Guangzhou 510650, China; nhxia@scbg.ac.cn

## Error in Figure/Table

1. In the original publication [1], there was a mistake in “Figure 4. Diagram of cis-acting elements in the promoters of *DhB21G011140*, *DhB31G002880*, *DhB31G019250*, *DhA19G015160*, *DhA19G013950*, and *DhB10G011540*.” as published. The mistake was that, due to a change in the SSRs in Table S4, the genes being located have changed. The corrected “Figure 4. Diagram of cis-acting elements in the promoters of *DhA24G003730.1* and *DhA15G005980.1*.” appears below. 

2. In the original publication [1], there was a mistake in “Table 2. Polymorphism indicators of 12 important materials were obtained using 34 SSR markers.” as published. Due to the changes in polymorphic SSRs in Table S4, deviations occurred in various genetic parameters in Table 2. The corrected “Table 2. Polymorphism indicators of 12 important materials were obtained using 28 SSR markers.” appears below. 

3. In the original publication [1], there was a mistake in “Figure 5. Cluster analysis of 12 materials based on SSR markers.” as published. Due to the changes in polymorphic SSRs in Table S4, partial alterations occurred in the clustering relationships among the 12 materials in Figure 5. The corrected “Figure 5. Cluster analysis of 12 materials based on 28 SSR markers.” appears below. 

4. In the original publication [1], there was a mistake in “Figure 6. DNA fingerprinting of 12 materials constructed based on 34 pairs of SSR primers.” as published. Due to the changes in polymorphic SSRs in Table S4, partial changes occurred in the DNA fingerprinting of the 12 materials in Figure 6. The corrected “Figure 6. DNA fingerprinting of 12 materials constructed based on 28 pairs of SSR primers. On the inner circle of the image is the name of the 28 SSRs.” appears below. 

5. In the original publication [1], there was a mistake in “Figure S3. The correlation between alleles and PIC values.” as published. Due to the changes in polymorphic SSRs in Table S4 and the alterations in genetic parameters in Table 2, partial deviations occurred in the scatter point positions and R^2^ values of the scatter plot in Figure S3; there was a mistake in “Figure S4. Cluster analysis of 15 sweet dragon bamboo germplasms based on SSR markers.” as published. Due to the changes in polymorphic SSRs in Table S4, partial alterations occurred in the clustering relationships among the 15 materials in Figure S4; there was a mistake in “Figure S5: Amplification results of core primer of 12 materials.” as published. As the DNA fingerprinting in Figure 6 revealed multiple pairs of core primer combinations, Table S8 was used to replace Figure S5; there was a mistake in “Table S4. Information on 34 pairs of polymorphic primers.” as published. Due to the mismatch between Column E and Column F, part of the data in this table are in disorder; there was a mistake in “Table S5. Genetic similarity coefficients among different materials.” as published. Due to the changes in polymorphic SSRs in Table S4, partial deviations occurred in the genetic similarity coefficients in Table S5; there was a mistake in “Table S6. DNA fingerprint information of the 12 materials.” as published. Due to the changes in polymorphic SSRs in Table S4, partial changes occurred in the DNA fingerprinting of the 12 materials in Table S6. Supplementary material to the original manuscript has also been updated.

## Text Correction

1. There was an error in the original publication [1]. Due to the changes in the number of polymorphic SSRs and gene mapping in Result 2.4, the relevant content in the abstract must be revised. 

A correction has been made to Abstract:

*Dendrocalamus brandisii* is a high-quality bamboo species that can be used for both bamboo shoots and wood. The nutritional components and flavors of *D. brandisii* from different geographical provenances vary. However, the unique biological characteristics of bamboo render morphological classification methods unsuitable for distinguishing them. Although the new cultivar ‘Manxie No.1’ has significant differences in the branch characteristics and the color of shoot sheaths compared with *D. brandisii*, precise genetic information at the molecular level is still lacking. This study identified 231,789 microsatellite markers based on the whole genome of *D. brandisii* and analyzed their type composition and distribution on chromosomes in detail. Then, using TP-M13-SSR fluorescence-labeling technology, 28 pairs of polymorphic primers were screened to identify the new cultivar ‘Manxie No.1’ and 11 different geographical provenances of *D. brandisii*. We also constructed DNA fingerprinting profiles for them. At the same time, we mapped two polymorphic SSRs to the CDS sequences of two genes of *D. brandisii*, among which SSR497 was mapped to *DhA15G005980.1*, which is related to plant growth and development processes, as well as hormone signal transduction pathways. The specific markers selected in this study can rapidly identify the provenances and the new cultivar of *D. brandisii*, and help to explore candidate genes related to some important traits.

2. There was an error in the original publication [1]. Due to the change in the number of polymorphic SSRs in Result 2.4, the number of polymorphic SSRs stated in the Introduction must be changed from 34 to 28.

A correction has been made to 1. Introduction, fourth paragraph:

At present, whole-genome SSR markers have been developed for only a few bamboo species in the genera *Phyllostachys* and *Guadua* and few related genetic analyses in bamboo, such as *Phyllostachys edulis* and *Guadua chacoensis*, have been conducted [15,22]. For the genus *Dendrocalamus*, there are only reports on the development of whole-genome-based SSR markers for *Dendrocalamus strictus* [23], genome survey sequencing-based SSR markers for *Dendrocalamus longispathus* [24], and RNA-seq-based SSR markers for *Dendrocalamus latiflorus* [25], whereas there have been no reports on the development of whole-genome SSR markers for other bamboo species in this genus. According to the existing literature, all studies mainly focus on different bamboo species. However, reports on the same bamboo species from different geographical provenances are scarce. This study was based on the genome of *D. brandisii*. SSR markers covering the whole genome were developed and located in different regions of chromosomes and genes. After the specific primers were obtained via agarose gel electrophoresis, 28 polymorphic primers were further screened using capillary electrophoresis to evaluate the polymorphism potential of the new cultivar ‘Manxie 1’ and 11 different geographical provenances, and their phylogenetic relationships were explained using cluster analysis. A fluorescent labeled TP-M13-SSR system was established to distinguish the new cultivar ‘Manxie No.1’ and different sources of *D. brandisii*, and their DNA fingerprintings were constructed, providing a simple method for their identification and protection.

3. There was an error in the original publication [1]. Due to the changes in the number of polymorphic SSRs and gene mapping in Table S4, the content related to the number of polymorphic SSRs and the gene mapping of SSRs in Result 2.4 needs to be revised.

A correction has been made to 2. Results, 2.4. Obtain 28 Pairs of Polymorphic Primers and Perform Gene Mapping on 2 of Them, first and second paragraphs:

We utilized the Primer3_core program for Perl language-designed batch primers for all microsatellite flank sequences, resulting in 20,228 pairs of primers. Then, we randomly selected 800 pairs of primers for synthesis, and combined PCR and agarose gel electrophoresis to preliminarily screen them; the results showed that 433 pairs of primers could amplify at least one band (Figure S2). We selected 72 pairs of specific primers that could only amplify one band to conduct TP-M13-SSR PCR amplification on ‘Manxie No.1’ and 11 different geographical provenances of *D. brandisii* materials. The capillary electrophoresis results showed that the results for the three individuals within each provenance population were consistent, so any one of the samples could be selected as a representative sample for that provenance population. And 28 pairs of primers (Table S4) exhibited polymorphism, with adjacent alleles of each marker differing by multiples of the repeat unit length, indicating that these 28 polymorphic SSR markers are effective and have conservation and transferability across cultivars and provenances.

In addition, we performed gene mapping on two SSRs (SSR236, SSR497) located on exons, which corresponded to *DhA24G003730.1* and *DhA15G005980.1*, respectively. Through homology alignment in the NCBI database, it was revealed that *DhA24G003730.1* encodes the dihydrolipoyllysine-residue acetyltransferase component 4 of the pyruvate dehydrogenase complex in chloroplasts. It is postulated to participate in the maintenance and renewal of the membrane structure within the chloroplast. *DhA15G005980.1* is a *DIVARICATA* transcription factor, which belongs to the *MYB* family. This gene is associated with various aspects of plant growth and development, and participates in regulating hormone signal transduction, non-biological stress resistance, and other processes. Notably, the promoters of both genes harbor cis-acting elements responsive to light, auxin, gibberellin, and methyl jasmonate (Figure 4). These elements likely play crucial roles in the transcriptional regulation of these genes in response to diverse environmental and hormonal cues, thereby modulating their physiological functions within the plant.

4. There was an error in the original publication [1]. Due to the changes in the number of polymorphic SSRs, the relevant genetic parameter values need to be changed accordingly in Result 2.5.

A correction has been made to 2. Results, 2.5. Genetic Diversity Analysis of ‘Manxie No.1’ and 11 Different Geographical Provenances of *D. brandisii* Materials Based on 28 Polymorphic SSRs, first paragraph:

Genetic diversity analysis was conducted on ‘Manxie No.1’ and 11 different geographical provenances of *D. brandisii* representative materials using 28 polymorphic SSR markers. A total of 28 polymorphic SSR markers amplified 81 alleles, with an average of 2.8929 alleles per marker. Among them, SSR394 amplified the largest number of alleles (Na = 6). In contrast, 14 markers, such as SSR52 and SSR18, amplified the fewest alleles (Na = 2). The range of Ne was 1.1803 to 4.0000, with an average of 2.0281 and an average Ho value of 0.5268, which was higher than the average He value of 0.4732. The average value of I was 0.7600 (range of 0.2868–1.4735). The PIC range of polymorphic information was 0.1411–0.7078, with an average value of 0.3863. There were seven pairs of primers with PIC < 0.25, indicating low polymorphism. The PIC of six primer pairs was greater than 0.5, which indicates that they possess high polymorphism (Table 2). The correlation graph between Na and PIC values shows that. as the number of alleles increased, the polymorphism of specific loci increased (Figure S3). The genetic parameters calculated based on 28 SSR markers indicate that there was a moderate degree of genetic diversity and variation rate between 11 different geographical provenances of *D. brandisii* and ‘Manxie No.1’.

5. There was an error in the original publication [1]. Due to the changes in the genetic similarity coefficients in Table S5, in the UPGMA tree constructed based on these coefficients in Result 2.6, the genetic relationships among some provenances have changed.

A correction has been made to 2. Results, 2.6. Cluster Analysis of 1 ‘Manxie No.1’ and 11 Different Geographical Provenances of *D. brandisii* Materials Based on 28 SSR Markers, first paragraphs:

Based on NTSYS v.2.10e software, the genetic similarity coefficient was calculated using the SM coefficient, and a UPGMA tree was constructed based on the genetic similarity coefficient. Similarly, as reported in Result 2.5, we conducted cluster analysis using a representative sample from each population. The analysis revealed that the new variety ‘Manxie No. 1’ exhibited a certain degree of genetic divergence compared with *D. brandisii*, with a mean genetic similarity coefficient of 0.3823, indicating a relatively distant genetic relationship. This finding is congruent with the fact that ‘Manxie No. 1’ is a superior variety obtained through artificial selection. In the case of *D. brandisii*, the samples from Lincang** (Linxiang District) and Yuxi City in Yunnan Province, China, demonstrated a more distant genetic relationship with the other nine provenances. The remaining nine provenances of *D. brandisii* could be approximately divided into two groups based on the clustering tree. The samples from Baoshan City and Dehong Dai and Jingpo Autonomous Prefecture (abbreviated as ‘Dehong’ in the following text), which are geographically adjacent to each other in Yunnan Province, China, constituted one group, with a genetic similarity coefficient of 0.7292 between them. In the other group, the samples from Chiang Mai, Thailand, formed a separate subgroup, while those from Mu Cang Chai County, Vietnam, Xishuangbanna Dai Autonomous Prefecture (the following text is abbreviated as ‘Xishuangbanna’) and Lincang* (Cangyuan County) in Yunnan Province, China, and Guangzhou in Guangdong Province, China, were grouped together. Among them, the samples from Mu Cang Chai County, Vietnam, and Xishuangbanna, China, exhibited the closest genetic relationship, with a genetic similarity coefficient of 0.8667. The samples from Pu’er City and Honghe Hani, and Yi Autonomous Prefecture (abbreviated as ‘Honghe’ in the following text), which are geographically adjacent to each other in Yunnan Province, China, constituted the third subgroup, with a genetic similarity coefficient of 0.7826 (Figure 5; Table S5). The clustering results indicate that there was a certain correlation between the genetic relationships of different geographical provenances of *D. brandisii* and their geographical distribution. However, it was not completely related to their geographical distribution. Further verification of the accuracy of the UPGMA tree was carried out through the Cophenetic values subroutine in the clustering program and the matrix comparison plot subroutine in the graphics program. The matrix correlation r value of the correlation test was 0.9424, proving that the reliability of the UPGMA tree was very high. 

6. There was an error in the original publication [1]. Due to the changes in polymorphic SSRs in Table S4, the fingerprinting patterns and core primers in Result 2.7 must be changed accordingly.

A correction has been made to 2. Results, 2.7. SSR Core Primers and DNA Fingerprinting of 1 ‘Manxie No.1’ and 11 Different Geographical Provenances of *D. brandisii* Materials, first paragraph:

In this study, primer SSR394 exhibited the highest number of producible bands (six bands), followed by SSR522 (five bands). These bands could be respectively assembled into five distinct banding patterns, which were utilized to discriminate five different materials. When the primer combinations of SSR522 and SSR394 were employed in conjunction with any one of SSR599, SSR100, SSR236, SSR527, or SSR39, a complete differentiation of the twelve materials was achieved. Consequently, these five combinations were each designated as a set of core primers. Moreover, four additional sets of primers were also authenticated as core primers. All nine sets of core primers are enumerated in Table S8. The DNA fingerprinting can be found in Figure 6 and Table S6. 

7. There was an error in the original publication [1]. Due to the change in the number of polymorphic SSRs in Table S4, the number “34” in the second paragraph of “3. Discussion, 3.2. Genetic Diversity within Bamboo Populations and Determination of Representative Samples for Various Populations of *D. brandisii*” and the first paragraph of “3.3. Genetic Diversity and Clustering of 1 ‘Manxie No.1’ and 11 Different Geographical Provenances of *D. brandisii*” was changed to “28”. In the second paragraph of “3.3. Genetic Diversity and Clustering of 1 ‘Manxie No.1’ and 11 Different Geographical Provenances of *D. brandisii*”, the genetic parameters were also revised according to Table 2. In the third paragraph, the core primers were modified according to Table S8.

A correction has been made to 3. Discussion, 3.3. Genetic Diversity and Clustering of 1 ‘Manxie No.1’ and 11 Different Geographical Provenances of *D. brandisii*, second and third paragraphs:

At present, there is relatively scarce information on the genetic diversity and population structure of bamboo germplasm resources. Due to the asexual reproduction of bamboo through rhizomes, it has low intra-species genetic diversity and genetic differences between populations [51]. Therefore, screening for markers with relatively high abundance and polymorphism for bamboo may be very difficult. Abreu developed and screened 7 polymorphic microsatellite markers for *Aulonemia aristulata* [52], and Vieira developed and screened 16 polymorphic microsatellite markers for the tropical woody bamboo Tribe Bambusae (Poaceae: Bambusoideae) [53]. Weixin developed and screened 20 polymorphic microsatellite markers for *Ph. Edulis* [32]. In this study, based on the ability of agarose gel to separate DNA fragments, 72 pairs of specific primers that can amplify only 1 band were initially screened from 800 pairs of primers, thus reducing the complexity and uncertainty in subsequent analyses. Then, based on capillary electrophoresis with higher resolution and sensitivity, 28 pairs of polymorphic primers were further selected from 72 pairs of specific primers that can amplify DNA fragments of different lengths in different samples. The differences in fragment lengths reflect the different number of motif repeats in SSR loci, thereby reflecting genetic differences between samples. Using these 28 pairs of polymorphic SSR primers, genetic diversity analysis was conducted on 12 samples (‘Manxie No.1’ and 11 different geographical provenances of *D. brandisii*). The PIC value takes into account the frequency and number of alleles and can comprehensively reflect the degree of polymorphism of the locus. For example, at an SSR locus, if there are multiple alleles and their frequency distribution in the sample is relatively uniform, the PIC value of the locus will be higher, indicating that the locus has high genetic diversity. In this study, the average PIC value of these 12 samples was 0.3863, ranging from 0.1411 to 0.7078, indicating that these samples had a moderate level of genetic diversity. This result provides important information for us to understand the genetic background of these samples. The moderate level of genetic diversity means that the samples had both a certain degree of genetic variability and relative stability. This genetic diversity may be due to the samples coming from different geographical provenances, experiencing different environmental choices, and genetic drift. On average, compared with other bamboo species using SSR molecular markers, the genetic diversity level of sweet dragon bamboo is higher than those of *Guadua inermis* and *Ph. edulis* [51,54] but lower than those of *G. angustifolia* in the Colombian coffee eco-region and *Kuruna debilis* [55,56].

Cluster analysis shows that the genetic diversity of *D. brandisii* and ‘Manxie No.1’ exhibits extensive variation. ‘Manxie No.1’ can be distinguished from different geographical provenances of *D. brandisii* and *D. hamiltonii,* and *D. asper* can also be distinguished from *D. brandisii*. Multiple primer combinations can distinguish 12 sweet dragon bamboo materials. However, clustering analysis shows that the genetic relationship between ‘Manxie No.1’ and *D. brandisii* is farther than that between *D. hamiltonii*, *D. asper* and *D. brandisii*. The possible reason for this result is that the number of microsatellite molecular markers is insufficient, leading to clustering bias. This situation can be improved by further screening polymorphic markers.

8. There was an error in the original publication [1]. Due to the change in the gene mapping of polymorphic SSRs in Table S4, the corresponding part in Discussion 3.4 must be changed accordingly.

A correction has been made to 3. Discussion, 3.4. Identification of Candidate Genes Based on Polymorphic SSRs, first paragraph:

This study performed gene localization on SSR236 and SSR497, and found that they were located in the exon regions of DhA24G003730.1 and DhA15G005980.1, respectively. DhA24G003730.1 encodes the dihydrothiolysine residue acetyltransferase component 4 of the pyruvate dehydrogenase complex in chloroplasts. A well-functioning pyruvate dehydrogenase complex is necessary for lipid synthesis, providing the necessary energy and material basis for the biosynthesis, maintenance, and renewal of thylakoid and chloroplast membranes [57–60], thereby ensuring the normal biological functions of chloroplasts. DhA15G005980.1, being a DIVARICATA transcription factor of the MYB family, has been demonstrated to play a significant and multifaceted role in plant growth and development, signal transduction, as well as in response to abiotic stresses. For instance, this transcription factor has been identified as essential for the determination of ventral petal identity in Antirrhinum majus [61], and it plays a pivotal role in establishing the floral dorsoventral asymmetry [62]. Moreover, it can negatively regulate salt stress in A. thaliana by integrating abscisic acid (ABA) signaling pathways [63]. The presence of cis-acting elements responsive to light, auxin, gibberellin, and methyl jasmonate within the promoters of both genes represents an intriguing discovery. These cis-acting elements are likely to act as regulatory switches, enabling the genes to respond to changes in the external and internal environment. In conclusion, the successful localization of SSR236 and SSR497 within DhA24G003730.1 and DhA15G005980.1, respectively, has opened up new avenues for exploring the chloroplast function, growth and development, plant hormone signal transduction, and resistance to abiotic stress in *D. brandisii*. 

9. There was an error in the original publication [1]. The method of selecting SSRs for the experiment in Method 4.3 has been modified.

A correction has been made to 4. Materials and Methods, 4.3. Screening for Specificity and Polymorphism of Primers, first paragraph:

We randomly selected 800 pairs of SSR primers covering 70 chromosomes for synthesis and used ‘Manxie No.1’ genomic DNA as a template for PCR amplification. When the size of the agarose gel electrophoresis band of the PCR amplification product met the expectation, it could be identified as a specific primer.

10. There was an error in the original publication [1]. In the “5. Conclusions” section, the content regarding the number of polymorphic SSRs and their gene mapping was revised.

A correction has been made to 5. Conclusions:

Based on the identification of a large number of SSRs from the genome of *D. brandisii*, 28 polymorphic microsatellite markers were screened and validated to quickly and accurately distinguish the new variety ‘Manxie No.1’ and *D. brandisii* from different geographical provenances. These markers help in understanding the distribution of genetic diversity and achieving the sustainable use of resources. At the same time, it provides reliable technical means for selecting varieties with excellent traits in horticultural production. Two polymorphic SSRs were mapped to the genes of *D. brandisii*. This provides candidate genes for studying its chloroplast function, growth and development, plant hormone signal transduction, resistance to abiotic stress, and other aspects. The identification technology based on microsatellite markers can be used as a part of product quality standards to standardize the production and circulation of horticultural products and improve the standardization and specialization level of the entire industry.

11. There was an error in the original publication. The title information of three attached figures and tables in the Supplementary Materials has been modified.

A correction has been made to Supplementary Materials:

The following supporting information can be downloaded at: https://www.mdpi.com/article/10.3390/plants13202910/s1, Figure S1: The difference in shoot sheath color and the number of main branches between ‘Manxie No.1’ and *D. brandisii*. On the left is *D. brandisii*, and on the right is ‘Manchier No.1’. (A) The color difference in shoot sheaths between the two. (B) The color difference in the number of branches between the two. Figure S2: Preliminary screening results of agarose gel electrophoresis with some primers. Figure S3: The correlation between alleles and PIC values. Figure S4: Cluster analysis of 15 sweet dragon bamboo germplasms based on SSR markers. Table S1: The number of perfect SSRs contained in compound SSRs and their respective proportions. Table S2: Connection sequence length of compound SSRs containing two perfect SSRs. Table S3: The number of motif repetitions of various types of perfect SSRs. Table S4: Information on 28 pairs of polymorphic primers. Table S5: Genetic similarity coefficients between every two of the 12 materials. Table S6: DNA fingerprint information of the 12 materials. Table S7: The PCR system and PCR program used in the TP-M13-SSR PCR method. Table S8: Nine sets of core primers for 12 materials.

## References

References [57–63] related to the genes where SSRs are mapped have been added, replacing the original [57–65]. Also, the original reference [69] related to the method of selecting SSRs for the experiment was deleted. With this correction, the order of some references was adjusted accordingly. 

The detailed information of the added [57–63] is as follows:57.Bohne, A.V.; Schwarz, C.; Schottkowski, M.; Lidschreiber, M.; Piotrowski, M.; Zerges, W.; Nickelsen, J. Reciprocal Regulation of Protein Synthesis and Carbon Metabolism for Thylakoid Membrane Biogenesis. *PLoS Biol.* **2013**, *11*, e1001482. https://doi.org/10.1371/journal.pbio.1001482.58.Benning, C. Mechanisms of Lipid Transport Involved in Organelle Biogenesis in Plant Cells. *Annu. Rev. Cell Dev. Biol.* **2009**, *25*, 71–91.59.Lin, M.; Oliver, D.J. The Role of Acetyl-Coenzyme A Synthetase in *Arabidopsis*. *Plant Physiol.* **2008**, *147*, 1822–1829. https://doi.org/10.1104/pp.108.121269.60.Tovar-Méndez, A.; Miernyk, J.A.; Randall, D.D. Regulation of Pyruvate Dehydrogenase Complex Activity in Plant Cells. *Eur. J. Biochem.* **2003**, *270*, 1043–1049.61.Perez-Rodriguez, M.; Jaffe, F.W.; Butelli, E.; Glover, B.J.; Martin, C. Development of Three Different Cell Types Is Associated with the Activity of a Specific *MYB* Transcription Factor in the Ventral Petal of *Antirrhinum Majus* Flowers. *Development* **2005**, *132*, 359–370. https://doi.org/10.1242/dev.01584.62.Lucibelli, F.; Valoroso, M.C.; Aceto, S. Radial or Bilateral? The Molecular Basis of Floral Symmetry. *Genes* **2020**, *11*, 395. https://doi.org/10.3390/genes11040395.63.Fang, Q.; Wang, Q.; Mao, H.; Xu, J.; Wang, Y.; Hu, H.; He, S.; Tu, J.; Cheng, C.; Tian, G.; et al. AtDIV2, an R-R-Type *MYB* Transcription Factor of *Arabidopsis*, Negatively Regulates Salt Stress by Modulating ABA Signaling. *Plant Cell Rep.* **2018**, *37*, 1499–1511. https://doi.org/10.1007/s00299-018-2321-6.

The authors state that the scientific conclusions are unaffected. This correction was approved by the Academic Editor. The original publication has also been updated.

## Figures and Tables

**Figure 4 plants-14-01416-f004:**
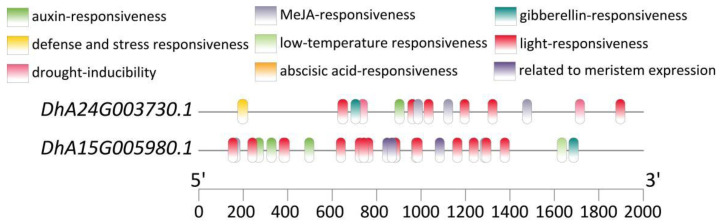
Diagram of cis-acting elements in the promoters of *DhA24G003730.1* and *DhA15G005980.1*.

**Figure 5 plants-14-01416-f005:**
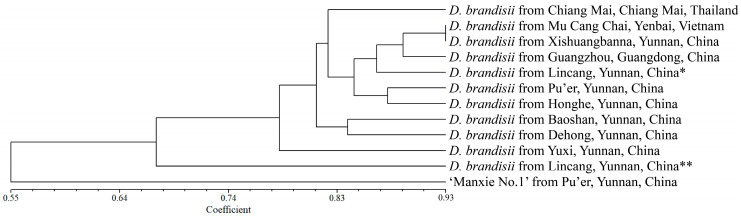
Cluster analysis of 12 materials based on 28 SSR markers. * represents Cangyuan County, Lincang City, Yunnan Province, China, and ** represents Linxiang District, Lincang City, Yunnan Province, China.

**Figure 6 plants-14-01416-f006:**
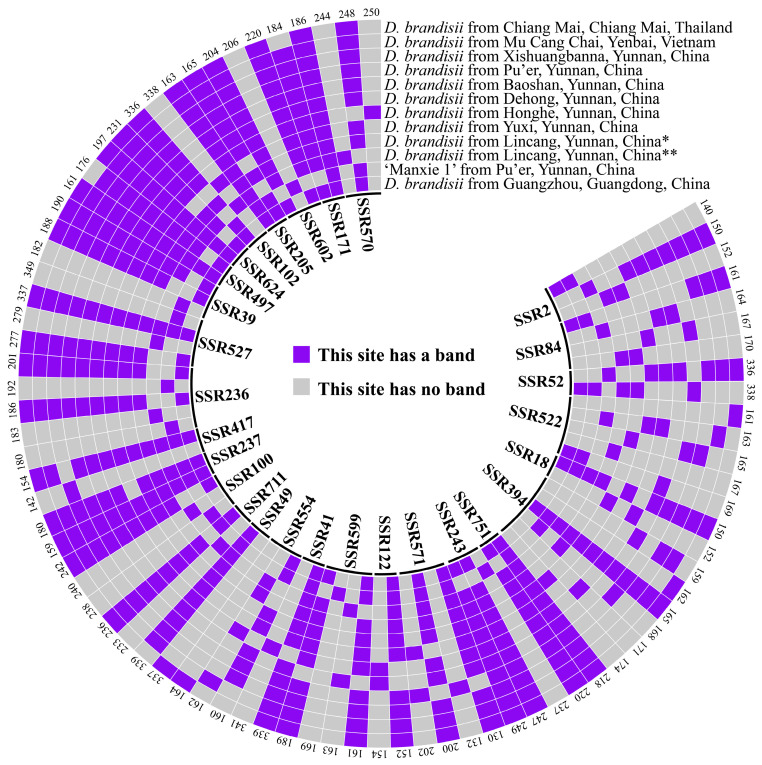
DNA fingerprinting of 12 materials constructed based on 28 pairs of SSR primers. On the inner circle of the image is the name of the 28 SSRs. The number on the outer circle of the image represents the size of all fragments that the corresponding SSR can amplify. Blue and gray, respectively, represent the presence or absence of fragments. * represents Cangyuan County, Lincang City, Yunnan Province, China, and ** represents Linxiang District, Lincang City, Yunnan Province, China.

**Table 2 plants-14-01416-t002:** Polymorphism indicators of 12 important materials were obtained using 28 SSR markers.

Primer ID	Na ^1^	Ne ^2^	I ^3^	Ho ^4^	He ^5^	PIC ^6^
SSR2	3.0000	2.0000	0.8676	0.4783	0.5217	0.4491
SSR84	4.0000	3.4286	1.3086	0.2609	0.7391	0.6589
SSR52	2.0000	1.9459	0.6792	0.4928	0.5072	0.3680
SSR522	5.0000	4.0000	1.4735	0.2174	0.7826	0.7078
SSR18	2.0000	1.3846	0.4506	0.7101	0.2899	0.2392
SSR394	6.0000	2.3415	1.2109	0.4022	0.5978	0.5436
SSR751	2.0000	1.9862	0.6897	0.4819	0.5181	0.3733
SSR243	3.0000	2.3415	0.9222	0.4022	0.5978	0.4788
SSR571	4.0000	2.5487	1.0618	0.3659	0.6341	0.5280
SSR122	2.0000	1.3846	0.4506	0.7101	0.2899	0.2392
SSR599	4.0000	2.7429	1.1219	0.3370	0.6630	0.5630
SSR41	2.0000	1.1803	0.2868	0.8406	0.1594	0.1411
SSR554	3.0000	2.1818	0.8877	0.4348	0.5652	0.4598
SSR49	2.0000	1.1803	0.2868	0.8406	0.1594	0.1411
SSR711	2.0000	1.1803	0.2868	0.8406	0.1594	0.1411
SSR100	3.0000	1.4118	0.5661	0.6957	0.3043	0.2723
SSR237	2.0000	1.9862	0.6897	0.4819	0.5181	0.3733
SSR417	2.0000	1.1803	0.2868	0.8406	0.1594	0.1411
SSR236	5.0000	2.7961	1.2015	0.3297	0.6703	0.5748
SSR527	4.0000	2.3415	0.9762	0.4022	0.5978	0.4832
SSR39	3.0000	2.3226	0.9184	0.4058	0.5942	0.4768
SSR497	2.0000	1.9459	0.6792	0.4928	0.5072	0.3680
SSR624	2.0000	1.9459	0.6792	0.4928	0.5072	0.3680
SSR102	2.0000	1.3846	0.4506	0.7101	0.2899	0.2392
SSR205	2.0000	1.9459	0.6792	0.4928	0.5072	0.3680
SSR602	3.0000	2.3415	0.9222	0.4022	0.5978	0.4788
SSR171	2.0000	1.9459	0.6792	0.4928	0.5072	0.3680
SSR570	3.0000	1.4118	0.5661	0.6957	0.3043	0.2723
Mean	2.8929	2.0281	0.7600	0.5268	0.4732	0.3863
St.	1.1333	0.6842	0.3268	0.1842	0.1842	0.1571

^1^ Observed number of alleles. ^2^ Effective number of alleles. ^3^ Shannon’s information index. ^4^ Observed heterozygosity. ^5^ Expected heterozygosity. ^6^ Polymorphic information content.
