# Supplementary figures and images for: Identification of New Cultivar and Different Provenances of Dendrocalamus brandisii (Poaceae: Bambusoideae) Using Simple Sequence Repeats Developed from the Whole Genome
Source: Plants (Basel). 2024 Oct 17;13(20):2910. doi: 10.3390/plants13202910 (PMC11511551; doi:10.3390/plants13202910)

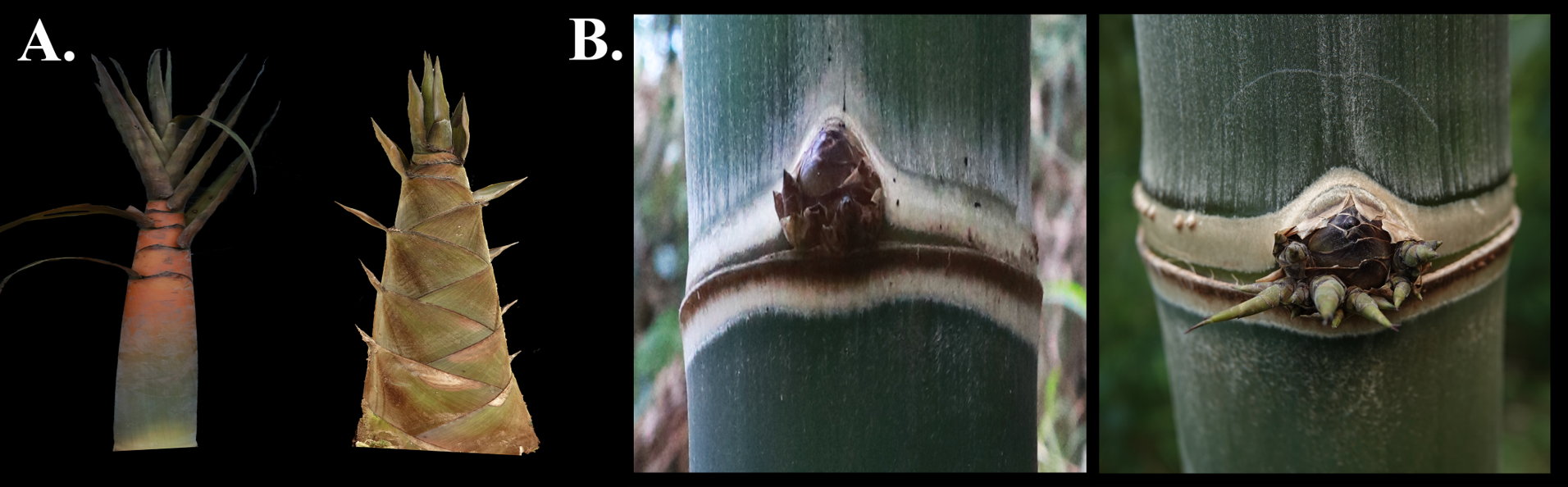

Supplement: Supplementary file 1 [file plants-13-02910-s001.zip › Figure S1 The difference in shoot sheaths color and the number of main branches between 'Manxie No.1' and D. brandisii.png]

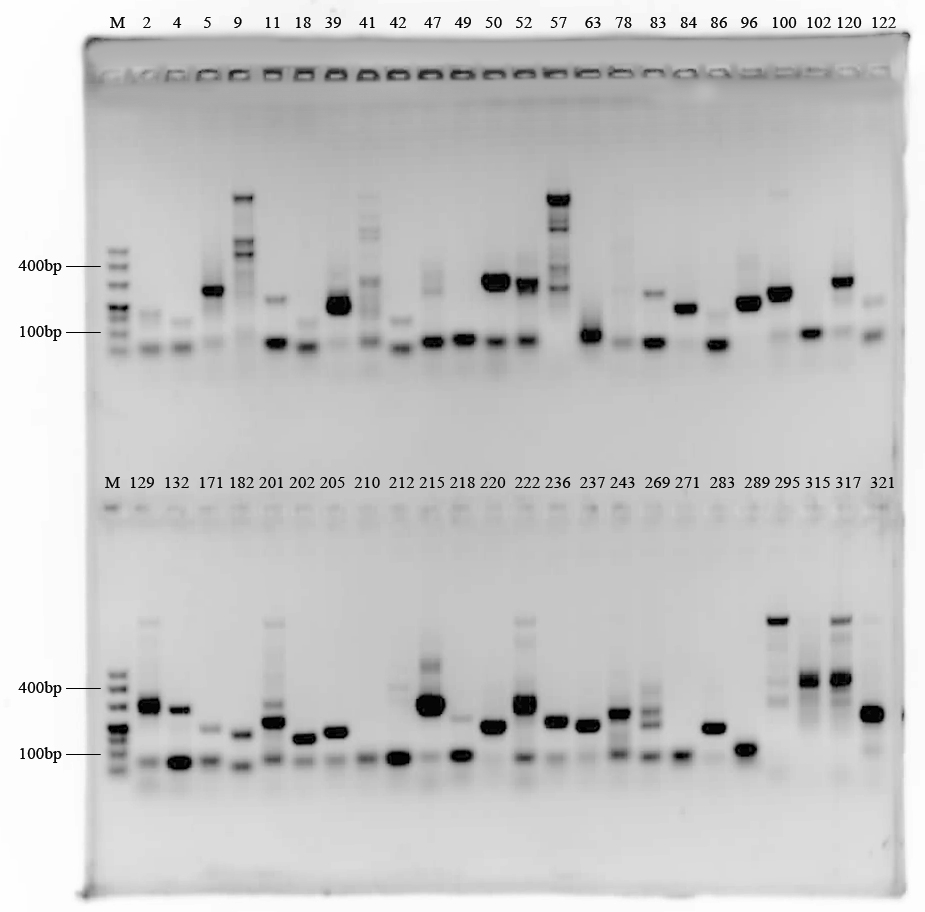

Supplement: Supplementary file 1 [file plants-13-02910-s001.zip › Figure S2. Preliminary screening results of agarose gel electrophoresis with some primers.png]

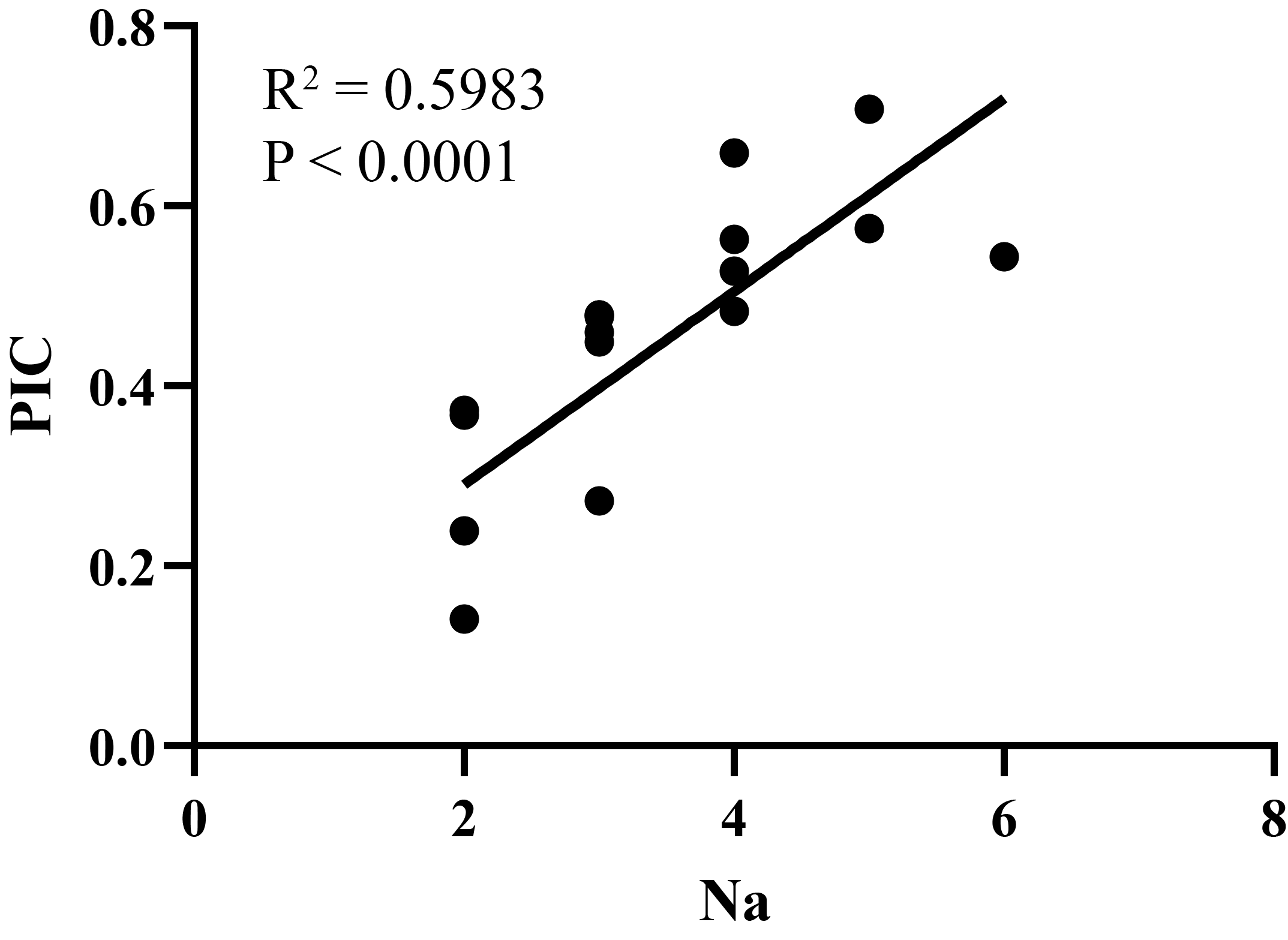

Supplement: Supplementary file 1 [file plants-13-02910-s001.zip › Figure S3. The correlation between alleles and PIC values.png]

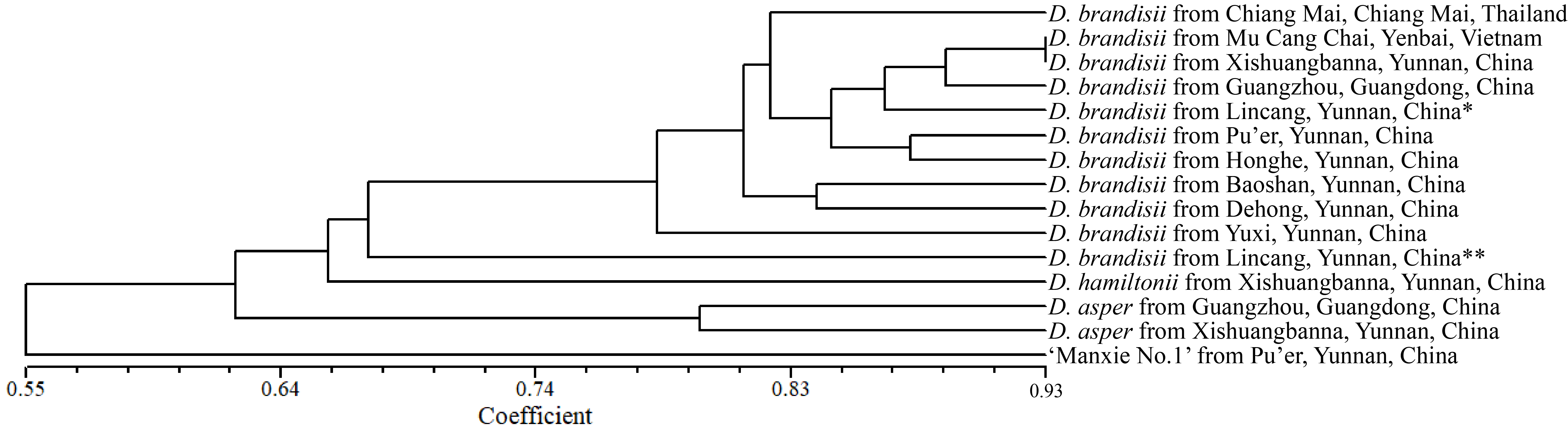

Supplement: Supplementary file 1 [file plants-13-02910-s001.zip › Figure S4. Cluster analysis of 15 sweet dragon bamboo germplasms based on SSR markers.png]
